# Severe chronic cerebral hypoperfusion induces microglial dysfunction leading to memory loss in APPswe/PS1 mice

**DOI:** 10.18632/oncotarget.7689

**Published:** 2016-02-24

**Authors:** Maude Bordeleau, Ayman ElAli, Serge Rivest

**Affiliations:** ^1^ Neuroscience Laboratory, CHU de Québec Research Center (CHUL), Department of Molecular Medicine, Faculty of Medicine, Laval University, Québec, Canada; ^2^ Neuroscience Laboratory, CHU de Québec Research Center (CHUL), Department of Psychiatry and Neuroscience, Faculty of Medicine, Laval University, Québec, Canada

**Keywords:** Alzheimer's disease, cerebral hypoperfusion, microglia, Gerotarget

## Abstract

Cerebral vasculature plays a key role in controlling brain homeostasis. Cerebral vasculature dysfunction, associated to irregularities in cerebral blood perfusion, has been proposed to directly contribute to Alzheimer's disease (AD) pathogenesis. More precisely, chronic cerebral hypoperfusion, which impairs brain homeostasis, was demonstrated to take place even before cognitive decline. However, the mechanisms underlying the implication of chronic cerebral hypoperfusion in AD pathogenesis remain elusive. Therefore, this study aims at investigating the role of severe chronic cerebral hypoperfusion (SCCH) in AD pathogenesis. For this purpose, SCCH was induced in young APPswe/PS1 in order to evaluate the progression of AD-like pathology in these mice. We observed that SCCH accelerated the cognitive decline of young APPswe/PS1 mice, which was associated with an increased amyloid plaque number in brain parenchyma. In addition, SCCH reduced the activity of extracellular signal-regulated kinases 1/2 (ERK1/2), which has been shown to play an important role in the adaptive responses of neurons. Importantly, SCCH impaired the function of microglial cells, which are implicated in amyloid-β (Aβ) elimination. *In vitro* approaches underlined the ability of a low-glucose microenvironment to decrease the general activity and phagocytic capacity of microglia. By using a new model of SCCH, our study unravels new insights into the implication of severe chronic cerebral hypoperfusion in AD pathogenesis, mainly by altering microglial cell activity and consequently Aβ clearance.

## INTRODUCTION

Alzheimer's disease (AD) is the most common form of dementia, which, according to current estimates, will be affecting up to 90 million patient worldwide by 2025 [1]. Amyloid-beta (Aβ) deposition and neurofibrillary tangle formation constitute the main pathological hallmarks of AD [2], which are respectively caused by aggregation of Aβ peptides and tau hyperphosphorylation in the brain. Previous reports have evidenced the existence of a pathological relationship between vascular risk factors and AD [3-7]. Consequently, it was proposed that vascular dysfunction associated to cerebral blood perfusion alterations might trigger downstream events that initiate the neurodegenerative cascade observed in AD [7, 8]. For example, reduced cerebral blood flow (CBF) has been associated with mild cognitive impairment (MCI), which has been proposed to be implicated in AD etiology [9-12]. Importantly, impaired CBF and glucose metabolism have been reported in AD patients [13], and in mouse models of AD [14]. More interestingly, impaired cerebral blood perfusion has been suggested to take place even before cognitive decline [15].

Cerebral vasculature comprises the blood-brain-barrier (BBB), which plays a major role in controlling brain homeostasis and cerebral Aβ elimination [16]. Recently, the two-hit vascular hypothesis incorporated BBB dysfunction associated to impaired cerebral blood perfusion as a major player in AD pathogenesis [16]. The hypothesis speculates that several risk factors affecting vascular functions, such as hypertension, diabetes, heart disease and cerebrovascular diseases, impair cerebral Aβ clearance across the BBB, consequently affecting regional brain microenvironment, and increasing cerebral Aβ accumulation and aggregation [16]. Indeed, many studies have reported higher Aβ levels following chronic cerebral hypoperfusion [17, 18], which triggers the formation of neurotoxic soluble Aβ oligomers [19]. Interestingly, Aβ has also been shown to profoundly affect the function of cells that form the neurovascular unit (NVU) [20]. The NVU is composed of vascular cells (endothelial cells and pericytes), glia cells (astrocytes and microglia), and neurons [20, 21]. The NVU plays a crucial role in maintaining brain homeostasis and CBF [20-24]. Recent reports have shown that chronic cerebral hypoperfusion (i.e. oligemia) impairs NVU function [10]. The implication of chronic cerebral hypoperfusion in inducing brain endothelial cell dysfunction is well documented [20, 25, 26]. In parallel, chronic cerebral hypoperfusion has been shown to alter astrocyte function, leading to abnormal axon-glia connections [27], characterized by the infiltration of astrocyte branches in neuronal cell bodies [28]. Importantly, the latter phenomenon is known to promote neuronal apoptosis [28]. Furthermore, several other studies have observed altered numbers of astrocytes [9, 29], neurons [26, 28, 30-32], microglia [28, 29, 32] and peripheral monocytes [33] following either chronic or acute hypoperfusion. Besides Aβ elimination across the BBB, the brain possesses several other sophisticated mechanisms involved in Aβ clearance, namely Aβ phagocytosis and degradation by microglia [34, 35]. However, Aβ clearance by microglia is impaired in the advanced stages of AD due to the formation of a stressful and unsuitable microenvironment [36]. Importantly, how chronic cerebral hypoperfusion may affect the function of microglia remains unknown [6].

As such, in the present study we aimed to investigate the implication of severe chronic cerebral hypoperfusion in AD pathogenesis. For this purpose, we developed a new model of severe chronic cerebral hypoperfusion (SCCH) (Supplementary Figure 1), which was induced in young APPswe/PS1 AD mouse model. Mice were used to investigate whether and how SCCH affects AD pathology with an emphasis on the role of microglia. Interestingly, SCCH accelerated the cognitive decline of young mice, which was accompanied by an increased number of Aβ plaques and altered microglial cell function. In parallel, we reported a reduced activity of extracellular signal-regulated kinase-1/2 (ERK1/2) pathway, which plays an important role in the adaptive responses of neurons [37]. The *in vitro* investigations revealed that the altered function of microglial cells might be due to an impaired glucose metabolism, as glucose lowering potently altered the activity and phagocytic capacity of microglia. Taken together, we report here that SCCH exacerbated AD-like pathology in young APPswe/PS1 mice by affecting the ability of microglia to naturally eliminate Aβ from the brain parenchyma.

## RESULTS

### SCCH worsens memory impairment in APPswe/PS1 mice without affecting motor capacity

**Figure 1:**
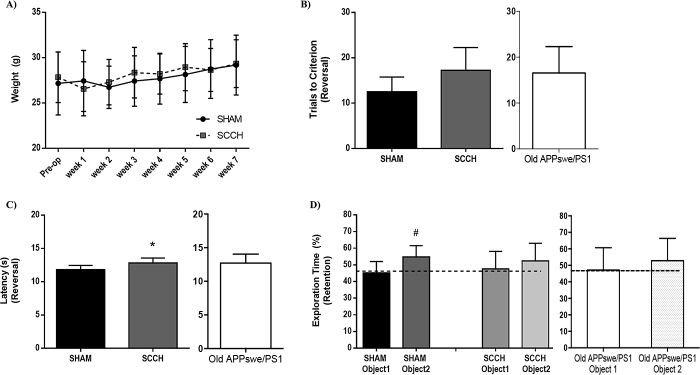
SCCH aggravates APP_swe_/PS1 cognitive deficits The surgery has no effect on animal weight **A.**. Old APPswe/PS1 mice were used as positive control for cognitive decline. Water t-maze **B.**,**C.** and novel object recognition tests **D.** were used to assess mouse cognitive function. SCCH slightly increases the number of trails **B.** and significantly increases the latency **C.** of mice in the water t-maze test, which were comparable to those of old mice. SCCH significantly reduces mouse capacity in recognizing the novel object during the NOR test, which also were comparable to those of old mice. Data are means ± SEM (*n* = 6-8 animals per group) * *P* < 0.05 compared to sham group; #*P* < 0.05 compared with sham object 1. Pre-op: Pre-operatory.

The weight of animals was monitored for up to 7 weeks after surgery, which did not reveal any difference between groups (Figure 1A). Water T-maze and novel object recognition (NOR) tests were performed 14 weeks after surgery to distinguish changes in hippocampal-dependent spatial memory and learning [38] from alterations in non-spatial memory [39], respectively. The non-spatial memory or recognition memory is based on the novelty paradigm which postulates a subject's preference to explore a new object rather than a familiar one [39]. In the reversal phase of water T-maze test, SCCH APPswe/PS1 mice needed more trials to complete the test (*P =* 0.0681) (Figure 1B) and had significantly (*P* = 0.0350) higher latency (Figure 1C) compared to sham APPswe/PS1 mice. This indicates a possible acceleration of spatial memory and learning deficits in response to SCCH. The cognitive decline in APPswe/PS1 mice is progressive and AD-like pathology worsens with age. Given the fact that changes in spatial memory and learning were mild, we used old APPswe/PS1 mice (10.5 months old) as a positive control, and we compared the cognitive profile of SCCH mice (7.5 months old) with these old mice to better assess spatial memory and learning decline of SCCH mice. Importantly, we found out that SCCH mice and old mice have similar cognitive profiles, confirming the acceleration of spatial memory and learning deficits of SCCH mice (Figure 1B, 1C). In the retention phase of the NOR test, SCCH mice showed a significant loss of the novelty paradigm compared to sham mice (*P* = 0.0243) (Figure 1D). Again, in order to better assess non-spatial memory decline of SCCH mice, we compared the cognitive profile of SCCH mice with old mice. Similarly, we found that SCCH mice and old mice have similar cognitive profiles, confirming the acceleration of non-spatial memory deficits of SCCH mice (Figure 1D). Sham and SCCH mice explored the objects 48.62-51.58% and 48.17-51.83% of the time, respectively.

Motor performance was assessed with open-field and asymmetry tests, which respectively define motor capacity and laterality tendency. Sham and SCCH mice behaved likewise for both tests (Supplementary Figure 2). During the open-field test, both groups showed equivalent distance traveled (sham: 33.3 ± 1.3 cm, SCCH: 27.4 ± 2.4 cm), immobile time (sham: 27.1 ± 4.2 sec, SCCH: 49 ± 14 sec), immobile episodes (sham: 16.7 ± 3.1, SCCH: 23.9 ± 4.2), maximum speed (sham: 0.320 ± 0.015 cm/s; SCCH: 0.327 ± 0.021 cm/s), as well as the number of total rotations (sham: 19.5 ± 0.3; SCCH: 16.5 ± 2.5), clockwise rotations (sham: 9.3 ± 1.2, SCCH: 8.5 ± 1.7) and anticlockwise rotations (sham: 10.2 ± 1.1, SCCH: 8.0 ± 1.5). In the asymmetry cylinder test, sham and SCCH mice used their right forepaw 50.8 ± 2.1% and 45.5 ± 2.7% of the time, respectively. Hence, chronic hypoperfusion did not elicit any significant asymmetry pattern. Altogether these results show that SCCH specifically affects spatial and non-spatial memory without altering motor abilities in APPswe/PS1.

### Memory loss in SCCH mice is associated with an increased number of parenchymal amyloid plaques

**Figure 2:**
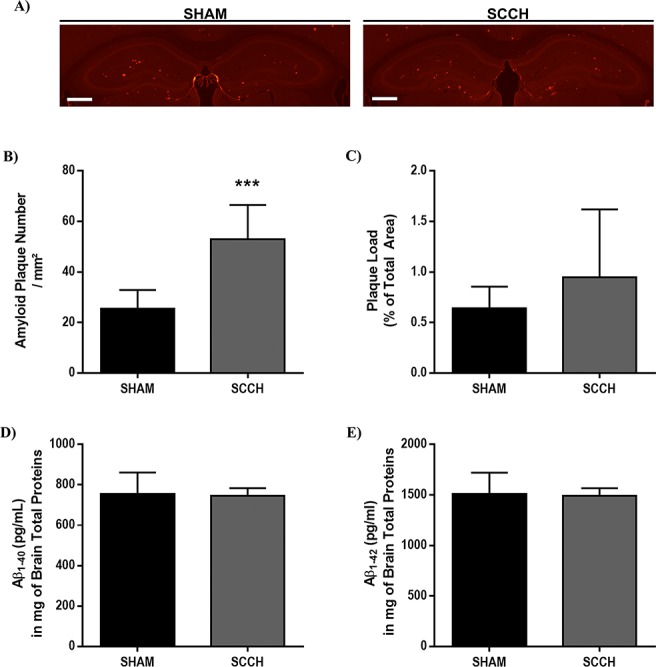
Number of Aβ plaques increases following SCCH without affecting soluble Aβ levels Aβ plaque number and size, and soluble Aβ peptide levels in the brain of APP_swe_/PS1 were assessed by immunofluorescence staining **A.**-**C.** and ELISA (**D**., **E**.), respectively. SCCH increases the number of Aβ plaques in the brain of mice **A.**,**B.** without affecting plaque load **C.**. The levels of soluble Aβ_1-40_ **D.** and soluble Aβ_1-42_ **E.** are not affected by SCCH. Data are means ± SEM (*n* = 6-8 animals per group, 3 sections per animal's brain for immunofluorescence staining) *** *P* < 0.005 compared with sham. Images were acquired with a 4X objective. Scale bar = 500μm.

Aβ deposition in brain parenchyma and vasculature constitutes one of the main hallmarks of AD [2]. In addition, soluble Aβ oligomers have been demonstrated to be particularly neurotoxic [19, 40]. Therefore, we first evaluated Aβ load in the hippocampus by using 6E10 immunostaining (Figure 2A-2C) and measured soluble Aβ_1-40_ and Aβ_1-42_ levels by ELISA (Figure 2D-2E). We decided to focus on the hippocampus since it is particularly vulnerable to chronic cerebral hypoperfusion [15, 23]. Stereological analysis of 6E10 staining revealed an increased number of Aβ plaques in the brain of SCCH mice compared to sham mice (*P* = 0.0008) (Figure 2A, 2B), whereas Aβ plaque load remained unchanged (Figure 2A, 2C). There was no difference in soluble Aβ_1-40_ (Figure 2D) and Aβ_1-42_ levels (Figure 2E) between the SCCH and sham groups. These results suggest that SCCH in APPswe/PS1 mice increases the number of Aβ plaques in the parenchyma without altering the levels of soluble Aβ.

### SCCH slightly increases the frequency of patrolling monocyte population

**Figure 3:**
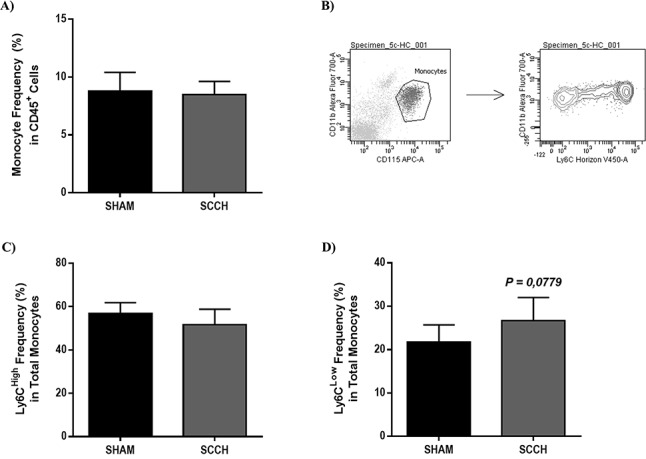
SCCH induces slightly increases patrolling monocyte subset frequency in blood circulation Frequency of total **A.**, inflammatory **C.** and patrolling monocytes **D.** were analyzed by flow cytometry in blood. Using a gating strategy based on Ly6C expression level **B.**, inflammatory and patrolling monocyte subsets were discriminated. SCCH does not affect the frequency of total monocyte frequency **A.** and inflammatory monocyte subset (Ly6C^High^) **C.**, in the blood of mice 14 weeks following surgery. SCCH slightly (*P* = 0.0779) increases the frequency of patrolling monocyte subset (Ly6C^Low^) in the blood of mice **D.**. Data are means ± SEM (*n* = 6-8 animals per group). Data were analyzed with standard two-tailed unpaired t- test's.

Monocytes are known to be recruited to vasculature following injury [41,42] and to participate in Aβ elimination [43-45]. Thus, we investigated the effect of SCCH on peripheral blood monocytes by using flow cytometry analysis. Leukocyte populations were gated with CD45^+^ in which CD11b^+^/CD115^+^ represented monocyte populations (Figure 3A). *Via* a gating strategy that uses Ly6C as a cell marker (Figure 3B), monocyte subsets were sorted into Ly6C^High^ (i.e. inflammatory monocytes) (Figure 3C) and Ly6C^Low^ (i.e. patrolling/anti-inflammatory monocytes) (Figure 3D). The frequency of total monocytes and Ly6C^High^ monocytes did not differ between SCCH and sham mice. However, the frequency of Ly6C^Low^ monocytes was slightly higher in SCCH mice (*P* < 0.0779) (Figure 3D). In order to understand the mechanisms underlying the profile of monocyte subset frequencies, we simultaneously analyzed the expression level of multiple key pro- and anti-inflammatory cytokines in the serum of mice, which included interleukin 1α (IL1α), IL1β, IL2, IL4, IL5, IL6, IL10, IL12, IL13, IL17A, granulocyte-colony stimulating factor (G-CSF) and granulocyte-macrophage-CSF (GM-CSF). SCCH significantly increased the protein expression level of IL5 in the serum of mice (*P =* 0.0143) without affecting the expression level of other cytokines (Supplementary Figure 3). IL5 is an anti-inflammatory cytokine, which has been reported to increase circulating monocytes [46]. The increased expression level of IL5 may explain, at least partly, the slight increase in the frequency of Ly6C^Low^ monocytes (anti-inflammatory monocytes). Taken together, it is conceivable to speculate that the slight increase of Ly6C^Low^ monocytes might be due to SCCH-induced cerebral vascular dysfunction accompanied by the increased production of IL5 [47-50]. These results suggest that monocytes were not implicated in Aβ processing in our experimental design.

Next, we examined BBB integrity following SCCH. BBB integrity was evaluated by assessing the extravasation of blood-derived IgG and albumin into brain, tight junction protein expression, and leukocyte infiltration into brain. Among BBB tight junction protein, we chose claudin-5, a major tight junction protein, which has been previously reported to decrease after bilateral carotid artery stenosis (BCAS) [26, 51]. SCCH did not trigger extravasation of IgG (Supplementary Figure 4A) or albumin (Supplementary Figure 4B) into brain parenchyma. Similarly, SCCH did not affect claudin-5 protein expression (Supplementary Figure 4C). Finally, SCCH did not induce the infiltration of leukocytes into brain, as shown by CD45 immunocytochemistry (Supplementary Figure 5). In all experiments, brain sections of mice subjected to permanent ischemic stroke were used as positive control.

### SCCH disrupts plaque coverage by microglia and alters microglial cell activation

**Figure 4:**
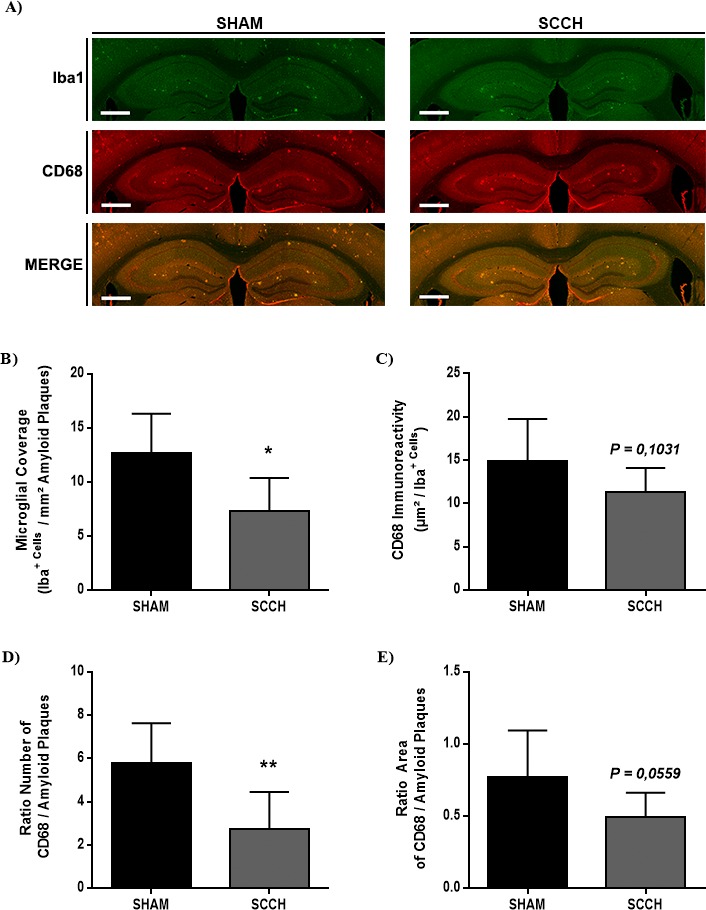
SCCH alters microglial cell function in APP_swe_/PS1 Microglial cell function was assessed by using immunofluorescence staining **A.**-**E.**. SCCH significantly decreases microglial coverage (Iba^+^) of Aβ plaques **A.**, **B.**, without affecting overall microglial activation (CD68^+^) **A.**, **C.**. SCCH significantly decreases the number of activated microglia associated to Aβ plaques **D.**, and slightly decreases (*P* = 0.0559) the area of Aβ plaques covered by activated microglia **E.**. Data are means ± SEM (*n* = 6-8 animals per group, 3 section per animal's brain for immunofluorescence staining). * *P* < 0.05, ** *P* < 0.01 compared with sham group. Images were acquired with 4X objective. Scale bar = 500μm.

After confirming that BBB integrity was not affected, we next assessed the effect of SCCH on microglial cell function, as these cells are implicated in Aβ clearance [36]. The coverage of Aβ plaques by microglia, and microglia activity were analyzed by using immunofluorescence staining against Iba1 CD68 and 6E10 (Figure 4). Interestingly, SCCH mice exhibited significantly lower microglial Aβ plaque coverage compared to sham mice (*P* = 0.0108) (Figure 4A, 4B). The number of CD68-immunoreactive microglia throughout the brain did not differ between groups (Figure 4C). However in the vicinity of Aβ plaques, the number of CD68-immunoreactive microglia significantly decreased (*P =* 0.0075) (Figure 4D) and the area covered by CD68-immunoreative microglia slightly decreased in SCCH mice (*P =* 0.0559) (Figure 4E). These results indicate that SCCH altered microglial cell activity specifically in the vicinity of Aβ plaques.

### Glucose metabolism impairment alters microglial cell function *in vitro*

**Figure 5:**
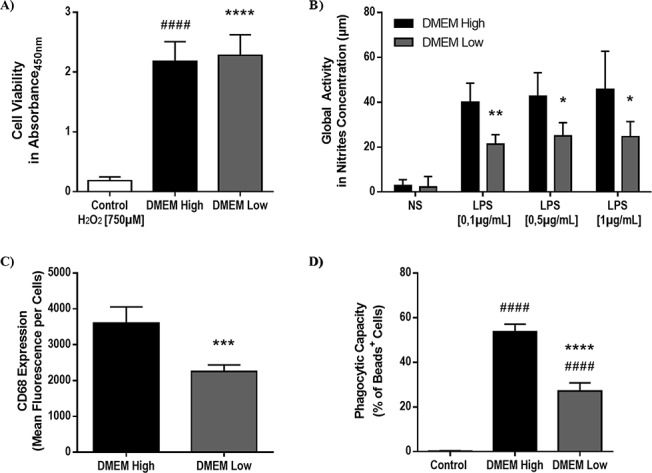
Low-glucose microenvironment alters microglial cell activity and phagocytic capacity Viability, global microglial cell activity and phagocytic capacity were investigated by using, XTT assay **A.**, Griess assay **B.** and flow cytometry **C.**, **D.**, respectively. Low-glucose microenvironment (i.e. DMEM-Low), maintained for 24 hours, does not alter BV2 cell (i.e. immortalized murine microglial cell) viability **A.**. BV2 cells exposed for 24 hours to 750 μM of H_2_O_2_ were used as a positive control for cell death **A.**. Low-glucose medium (i.e. DMEM-Low) significantly decreases global microglial cell activity **B.**, activation **C.** and phagocytic capacity **D.**. BV2 cells incubated on ice were used as negative control for the phagocytosis assay **D.**. DMEM-High (4500mg/L glucose) and DMEM-Low (1000mg/L glucose) medium were used. Data are means ± S.D. (*n* = 5 experiments per condition, 4 replicates per experiment for viability assay; *n* = 6 experiments per condition, 3 replicates per experiment for Griess assay; *n* = 5-6 samples per condition for flow cytometry). * *P* < 0.05, ** *P* < 0.01, *** *P* < 0.005, **** *P* < 0.0001 compared with DMEM High group; ^####^ *P* < 0.0001 compared with control.

We hypothesized that the altered immunoreactivity of microglia in the vicinity of Aβ plaques may be due to glucose metabolism impairment associated to chronic cerebral hypoperfusion. To verify this hypothesis, we used an *in vitro* approach by maintaining the murine immortalized microglial cell line BV2 in a low-glucose microenvironment that reflects, to some level, glucose metabolism impairment *in vivo.* By using an XTT assay that assesses cell survival, we demonstrated that the low-glucose medium (DMEM-Low) had no effect on cell viability compared to normal-glucose medium (DMEM-High) (Figure 5A). In addition, DMEM-Low had no effects on cell proliferation (DMEM-High, 3 h: 1.57 ± 0.05, DMEM-Low, 3 h: 1.59 ± 0.10, DMEM-High, 24 h: 2.18 ± 0.15, DMEM-Low, 24, h: 2.28 ± 0.15). These results suggest that a low-glucose microenvironment directly affects microglial cell viability and proliferation.

Next, we assessed microglial cell global activity, activation and phagocytic capacity. Global activity was determined by nitrite production following BV2 exposure to increasing concentrations of lipopolysaccharide (LPS), a potent microglial cell activator. Interestingly, BV2 cells maintained in DMEM-Low produced significantly less nitrites than cells maintained in DMEM-Low in presence of 0.1 μg/mL LPS (*P =* 0.0022), 0.5 μg/mL (*P =* 0.0112) and 1 μg/mL (*P =* 0.0321) (Figure 5B). We also examined microglial cell activation by quantifying CD68-immunoreactive BV2 cells by flow cytometry (Figure 5C). Interestingly, we found that DMEM-Low significantly decreased CD68-immunoreactivity compared to DMEM-High (*P =* 0.0003) (Figure 5C). Finally, we examined the implication of low-glucose microenvironment on microglial cell phagocytic capacity. For this purpose, we assessed the internalization of fluorescent beads by BV2 cells maintained in either DMEM-High or DMEM-Low for 24 h by flow cytometry (Figure 5D). Importantly, the internalization of fluorescent beads significantly decreased when BV2 were maintained in DMEM-Low (*P* < 0.0001) (27.05 ± 1.51 %) compared to DMEM-High (53.73 ± 1.35 %) (Figure 5D). These results suggest that impaired glucose bioavailability associated to an impaired glucose metabolism alters microglial cell function *in vitro*.

### SCCH reduces the activity of ERK1/2 signaling pathway

**Figure 6:**
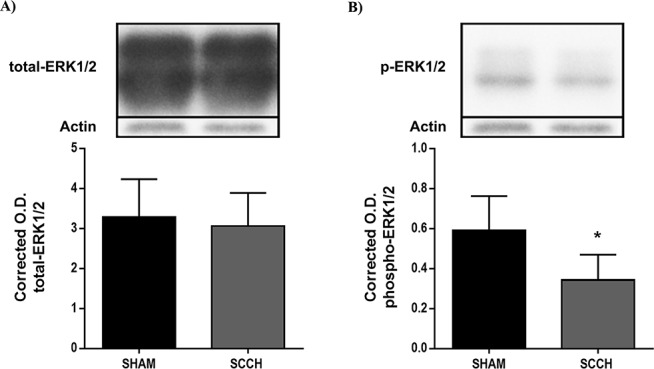
SCCH decreases ERK1/2 pathway activation Western blot analysis was used to assess total **A.** and phosphorylated **B.** ERK1/2, which were corrected with β-actin expression level. SCCH does not affect total ERK1/2 expression **A.**, but decreases ERK1/2 phosphorylation (i.e. activation) **B.**. Data are means ± SEM (*n* = 5-7 animals per group). * *P* < 0.05 compared with sham.

Next we examined the effect of SCCH on neuronal function and survival. For this purpose, structural changes in hippocampal neurons were investigated and the activity of ERK1/2 pathway, which is involved in the function and adaptive responses of neurons [37]. FJB staining was used to assess neuronal death, whereas Nissl staining was used to evaluate the structural changes of hippocampal neurons. SCCH did not affect neuronal survival (Supplementary Figure 6A), and did not alter neuronal structure in the hippocampus (Supplementary Figure 6B). Interestingly, SCCH significantly reduced ERK1/2 phosphorylation (i.e. activity) (*P =* 0.0161) (Figure 6). These results indicate that SCCH did not trigger neuronal death, but possibility triggered subtle neuronal dysfunction.

## DISCUSSION

The study aimed essentially at investigating the implication of SCCH on the progression of AD, which was motivated by the fact that most AD patients present several chronic cerebral perfusion problems. For this purpose, we established a new mouse model by inducing SCCH in young APPswe/PS1 mice. These mice allowed us to investigate AD-like pathology progression. We found that SCCH increased the number of Aβ plaques and worsened the cognitive decline of mice. These observations were accompanied by a decreased activity of microglia, which are implicated in Aβ elimination *via* phagocytosis, in the vicinity of Aβ plaques. This reduced activity *in vivo* may have been induced by low-glucose bioavailability in the brain following SCCH, as *in vitro* experiments showed that low-glucose microenvironment altered the activity and phagocytosis capacity of microglia. However, further studies are warranted to fully address the role of glucose metabolism impairment in microglial cell dysfunction *in vivo*.

SCCH offers several advantages compared to other approaches used to induce severe cerebral hypoperfusion by being more clinically relevant and by initiating cerebral hypoperfusion without inducing ischemic lesions. For instance in this model, chronic cerebral hypoperfusion is associated to a cardiac arrest simulation by transiently occluding the left common carotid artery without inducing ischemic lesions, reflecting evidence that supports a strong and causal association between cardiovascular diseases with cognitive decline in AD [52]. On the other hand, the widely used one vessel occlusion model (1VO) [25], which initiates a mild chronic cerebral hypoperfusion, is not associated to direct cardiac dysfunction. In addition, the other widely used transient bilateral carotid artery ligation (BCAL) model, which consists on clipping both arteries for few minutes, or BCAS, which is induced by narrowing carotid arteries using microcoils, initiate both a severe reduction in cerebral blood perfusion accompanied by the generation of ischemic lesions [26, 53].

In this study, memory assessment showed that SCCH aggravated the cognitive decline of young APPswe/PS1 mice, which was not accompanied by any motor deficit, indicating that SCCH affects essentially memory. Although clinical studies suggested that chronic cerebral hypoperfusion may affect cognitive function [15], only few studies examined the implication of chronic cerebral hypoperfusion in AD mouse models. In one study, mild chronic cerebral hypoperfusion induced by 1VO has been shown to exacerbate memory deficits in APPswe/PS1 mice [11, 33]. In addition, another study showed that chronic cerebral hypoperfusion induced by BCAS model exacerbated learning impairment in J20/APP AD mouse model [54].

The detrimental effects of SCCH on AD-like pathology in APPswe/PS1 mice were not associated to BBB breakdown or neuronal death, suggesting that the cognitive decline is probably caused by neuronal dysfunction. Indeed, it has been previously shown that oligemia associated to chronic cerebral hypoperfusion triggers neuronal dysfunction without necessary inducing neuronal death [17, 33].

In this study, SCCH increased the number of Aβ plaques in APPswe/PS1 mice without inducing significant changes in Aβ plaque size and soluble Aβ levels. Cerebral hypoperfusion affects Aβ processing. Upon severe oligemia induced by hypoxia, Koike et al (2010) reported an enhanced expression of β-secretase protein, an enzyme involved in Aβ processing, which was accompanied with an increased accumulation of soluble Aβ [17, 19]. In contrast with this hypoxic model [17], our results did not show any changes in soluble Aβ levels. The formation of Aβ plaques is the results of time-dependent soluble Aβ aggregation. Several studies have shown that Aβ plaques and soluble Aβ species do not always follow similar patterns in response to different treatments in AD mouse models. However, our group has previously demonstrated a positive correlation between the number of Aβ plaques and cognitive decline in APPswe/PS1 mice subjected to mild chronic cerebral hypoperfuison [33].

Cerebral vasculature injury is known to affect the dynamics of circulating monocytes [49, 50]. More precisely, Ly6C^High^ monocytes are recruited to injury site to phagocyte cell debris [42, 51, 55], whereas Ly6C^Low^ monocytes are recruited to the injured vasculature to promote vascular remodeling [49, 51, 56]. In our study, SCCH did not affect the frequency of total monocytes and Ly6C^High^ monocytes, which may be due to the absence of ischemic lesions. However, we noticed a slight increase in the frequency of Ly6C^Low^ monocytes. SCCH increased the protein level of the anti-inflammatory cytokine IL5, an anti-inflammatiry cytokine, which has been reported to increase circulating monocytes [46]. The increased protein level of IL5 in mice serum may partly explain the slight increase of anti-inflammatory monocyte subset (i.e. Ly6C^Low^ monocytes) frequency in blood circulation. However, these changes are subtle and do not seem to be implicated in Aβ processing.

The efficacy of microglia in eliminating Aβ decreases over time in AD due to the formation of a stressful microenvironment [36]. In this study, we showed that SCCH deeply affected microglial cell function, by reducing their activation and recruitment to Aβ plaques. Given the fact that microglia are involved in Aβ phagocytosis and elimination within Aβ plaques [35], the reduced number of activated microglia in the vicinity of Aβ plaques may account for the increased number of Aβ plaques. Glucose is the main source of energy for brain cells [57]. Glucose demand and consumption by microglia increase during activation and phagocytosis [58]. Therefore, a low-glucose microenvironment cannot support microglial cell metabolic needs. Importantly, chronic cerebral hypoperfusion has been reported to be systemically accompanied by an impaired glucose metabolism in the affected brain regions. We hypothesized here that SCCH may have impaired glucose metabolism, and thus consequently altered the microglial cell capacity in phagocyting Aβ. However, due to the complexity of addressing this issue *in vivo*, we used an *in vitro* approach. Low-glucose microenvironment significantly decreased the metabolic activity of microglia, which was accompanied by an altered microglial activation and phagocytic capacity. However, further studies are warranted to address the implication of glucose metabolism in alerting microglial cell activation and phagocytic capacity and the impact on Aβ processing *in vivo*.

Finally, we found that SCCH reduced the activity of ERK1/2 pathway. The biological significance of this result is elusive. However, due to its role in neuronal function and adaptive responses, it is conceivable to speculate that ERK1/2 pathway reduced activity may reflect neuronal dysfunction in the brain of APPswe/PS1 mice subjected to SCCH.

Altogether, our findings suggest that SCCH aggravates AD-like pathology in APPswe/PS1 mice by alerting microglial cell function. Several therapeutic strategies have been used to stimulate microglia for enhancing Aβ elimination. However, all these studies used transgenic mice that do not present cerebral perfusion problems, which may have affected microglial function. As such, we believe that future studies aiming to develop new strategies that target microglial cell function should take into consideration the implication of chronic cerebral hypoperfusion in order to achieve powerful therapeutic interventions.

## MATERIALS AND METHODS

### Animals with severe chronic cerebral hypoperfusion

All protocols were performed according to the Canadian Council on Animal Care guidelines, under supervision by the Laval University Animal Welfare Committee. Mice were acclimatized to standard laboratory cycles of 12 h light/dark (on at 7:00 h and off at 21:00 h). Adult male APPswe/PS1 transgenic mice expressing a doubly mutated version of human amyloid precursor protein (APPswe) as well as human presenilin 1 (A246E variant) were acquired from the Jackson Laboratory (Bar Harbor, ME). All mice had a C57BL/6J background.

Four months-old APPswe/PS1 mice were subjected to either sham or SCCH surgery. Mice were anesthetized for 5 min with 3.0% isoflurane (Abbvie, Chicago, IL), and anesthesia was further maintained using 2.5% isoflurane for ∼15 min. Lidocaine was injected locally before performing incisions. Midline cervical incision was performed and both common carotid arteries were then exposed. The right common carotid artery was permanently ligated with 6.0-mm non-absorbable silk thread as described in Pimentel-Coelho et al. (2013) [33]. After 2 min, a pressure of 15 g/mm^2^ was applied on the left common carotid artery with a vessel clamp (MORIA Inc, Doylestown, PA) during 15 sec (Supplementary Figure 1). Midline incision was closed using 6.0-mm silk suture thread, and mice were awakened using a heating pad. Buprenorphine was administered 12 h post operation. Body mass time course after surgery was monitored for eight weeks. Mice were kept for 17 weeks after surgery. Sham surgeries were performed by exposing both arteries and by wrapping a silk thread around the left artery without tightening to not obstruct the blood flow.

Fourteen weeks after surgery, mice were 7.5 months old, behavioral tests were performed. Three weeks later, mice were anesthetized with ketamine/xylazine and sacrificed by a transcardiac perfusion with normal saline solution (0.9% (w/v) NaCl). Just before sacrificing mice, cardiac blood was collected in EDTA-coated vials (Sarstedt, Newton, NC) for subsequent analysis. Brains were removed and split in two halves at the origin of the hippocampus (−1.0 mm from the bregma). The posterior part of the brain, along with the midbrain, was post-fixed with 4% (w/v) paraformaldehyde (PFA; Electron Microscopy Sciences, Hatfield, PA, USA) in 0.1 M phosphate buffer (pH = 7.6) for 48 h and immersed in 20% (w/v) sucrose/4% (w/v) PFA overnight. PFA fixed-brain samples were cut into 25-μm coronal sections with a microtome (Leica SM 2000R, Leica Microsystems Inc., Concord, ON). Tissues were stored at −20°C in a cryoprotectant solution (0.052 M sodium phosphate buffer (pH = 7.3) containing 5.37 M ethylene glycol and 2.71 M glycerol), which was used for immunofluorescence, Fluoro Jade-B (FJB) staining, Nissl body staining, immunohistochemistry and claudin-5 blot. Immunohistochemistry and FJB staining were carried out in parallel in stroked animals as positive controls for loss of BBB integrity and monocyte infiltration. The anterior part of the brain was frozen in dry ice and was used to perform all molecular analyses (*vide infra*).

### Behavior tests

Behavior tests were carried out 14 weeks post-surgery. All behavior tests were performed during the lights-off phase of the day and used 10,5 months old mice as a positive control. The behavioral experimenter was blinded to the surgery status of the animals.

### Water T-maze

Hippocampal-dependent spatial learning and memory were determined by using the water T-maze test as previously described [37]. Mouse ability to find a submerged platform in a water-filled fiberglass pool (stem length, 64 cm; arm length, 30 cm; width, 12 cm; wall height, 16 cm) was evaluated. Before the test, the escape platform (11 cm x 11 cm) was placed randomly at the end of one of the arms and submerged to a 1-cm depth. Mice were put in stem enclosure and allowed to explore the maze until the animal escaped to the platform. If the mouse did not find the submerged platform within 60 sec, it was gently maneuvered towards it. The animals stayed on the platform for 20 sec. During the learning and reversal phase, the time and number of trials required to reach the platform were noted. The criterion of positive learning was determined as successfully reaching the platform upon 5 consecutive trials; otherwise, mice had 48 trials at their disposal to achieve learning. Forty-eight hours later, the reversal-learning phase was assessed by placing the submerged platform at the opposite end of the maze

### NOR

The NOR test consisted of training and testing phases in a rectangular open field made of clear plexiglas of the same size as their home cage (width, 18 cm; length, 28 cm; height, 12 cm). All measures were taken so that mice do not see the experimenter while the test took place. During the training phase, two identical objects were presented to mice, which were allowed to inspect both of them for 5 min. Mice with an exploration period lasted less than 10 sec per object were considered unsuitable and were not used for further analysis. Between each trial, objects were thoroughly cleaned to minimize olfactory cues. After 3 h, the retention phase was performed. Mice were left in the experimental arena for 5 min in the presence of a familiar object along with a new object of comparable size, texture and shape. For half of the animals, the new object was presented on the right-hand side and for the remaining half, on the left-hand side. A digital camera was mounted on the ceiling above the arena and connected to a computer equipped with a video tracking system (ANY-maze, Stoelting Co., Wood Dale, IL), which objectively monitored and quantified movements. Object exploration was defined as touching the object or pointing the snout towards it at a distance < 2 cm. The recognition value represents ratio of the time spent exploring the object on the total time allowed.

### Asymmetry cylinder test

Mice were subjected to a single 5-min session within a glass cylinder (diameter, 20 cm; height, 30 cm). An angled mirror was placed behind the cylinder in order to visualize limb use and movements from all angles. During the session, forelimb asymmetry was assessed by scoring independent weight-bearing contacts of the right or left paw on the cylinder wall. The percentage of right and left touches was calculated relative to the total number of contacts.

### Open field

Mice were placed in a rectangular open-field arena for 5 min as previously described [59] and equipped with a video tracking system (side view). The distance traveled, the frequency and time of the motionlessness episode, the frequency and direction of rotation (clockwise or counterclockwise) as well as maximum speed reached were recorded. The time spent and entries in the different parts of the arena (center, interspace and periphery) were also recorded. After each trial, fecal waste was removed, and the floor of the arena was cleaned with a damp cloth and then dried.

### Soluble Aβ_1-40_ and soluble Aβ_1-42_ ELISA

Soluble Aβ_1-40_ and soluble Aβ_1-42_ ELISAs were performed according to manufacturer protocol (EMD Millipore, Billerica, MA). Contralateral and ipsilateral hemispheres were split and homogenized in 750 μL of lysis buffer containing a protease inhibitor cocktail (EMD Millipore) and 1% (v/v) phosphatase inhibitor cocktail 3 (Sigma-Aldrich, St. Louis, MO) with a hand-held homogenizer (Bio-Gep Series Pro200, Pro Scientific, Oxford, CT). A sample of the homogenized brain solution was collected for western blot analysis. The remaining solution was mixed at 4°C for 2 h. Brain homogenates were centrifuged for 10 min x 1300 rpm at 4°C and the supernatant was collected. Fifty μL of the diluted samples (1:10) and standards (16-500 pg/mL) were loaded along with 50 μL of antibody conjugate per well into 96-well ELISA microplates. Samples were mixed for 5 min at 4°C and then stored overnight at 4°C without agitation. Wells were rinsed five times with washing buffer prior to incubation with conjugate-enzyme for 30 min at room temperature (RT). Wells were rinsed again before substrate addition. ELISA microplates were incubated for 30-35 min at RT and the binding reaction was ended by adding a stop solution containing 0.3 M HCl. Soluble Aβ_1-40_ and soluble Aβ_1-42_ levels were measured by reading the absorbance at 450 nm (590 nm for correction) using a microtiter plate reader (SpectraMax 340PC, Molecular Devices, Sunnyvale, CA) and analyzed using the SOFTmax Pro3.1.1 software (Molecular Devices).

### Cytokine ELISA

The expression level of multiple cytokines was profiled in the serum of mice by using the Multi-Analyte ELISArray kit according to manufacturer protocol (Qiagen Inc., Toronto, ON). Twelve common major mouse cytokines were analyzed, which included IL1α, IL1β, IL2, IL4, IL5, IL6, IL10, IL12, IL13, IL17A, G-CSF and GM-CSF. Briefly, 50 μL of diluted serum samples (1:3) and negative/positive control standards were loaded per well into 96-well ELISA microplates and incubated for 2 at RT. Next 100 μL of detection antibody dilution was added to each well and incubated for 1 h at RT. Wells were rinsed five times with washing buffer prior to incubation with Avidin-HRP for 30 min at room temperature (RT). Wells were rinsed again and 100 μL of development solution were added in each well and incubated for 15 min at RT. The reaction was ended by adding 100 μL of stop solution containing. Cytokine protein levels were measured by reading the absorbance at 450 nm (correction at 570 nm) the using a microtiter plate reader (SpectraMax 340PC, Molecular Devices) and analyzed using the SOFTmax Pro3.1.1 software (Molecular Devices).

### Immunofluorescence staining

Brain sections were stained using a free-floating technique. Tissues were rinsed 3 times for 15 min in potassium phosphate buffered saline (KPBS) containing 22 mM K_2_HPO_4_, 3.3 mM KH_2_PO_4_ and 140 mM NaCl. Sections were then permeabilized and blocked in 6.78 mM Triton X-100, 1% (w/v) BSA (Sigma-Aldrich) and 4% (v/v) goat serum (Cedarlane, Burlington, ON) for 20-30 min. Tissues were incubated overnight at 4°C with the primary antibody in 0.5X blocking solution. We used mouse anti-Aβ monoclonal antibody (6E10) (Wako Chemicals, Richmond, VA), rabbit anti-ionized calcium binding adaptor molecule 1 (Iba1) antibody (Wako Chemicals) and rat anti-CD68 (AbD Serotec, Kidlington) as primary antibodies. Free-floating slices were next rinsed in KPBS and incubated with the secondary antibody for 2 h at RT. Tissues were incubated with Cy3-conjugated goat anti-mouse antibody (Jackson ImmunoResearch, West Grove, PA), Alexa 488-conjugated goat anti-rabbit antibody (Life Technologies, Burlington, ON) or Cy3-conjugated goat anti-rat antibody (Jackson ImmunoResearch). Brain sections were washed and counterstained with 2 μg/mL DAPI (Life Technologies) for 20 min at RT. Sections were rinsed, mounted onto SuperFrost slides and dried under vacuum for at least 3 h. Brain mounted slides were hydrated and overlaid with coverslips using iFluoromount-G anti-fading medium (Electron Microscopy Sciences).

For cellular stereological analysis, Iba1 was co-stained with either 6E10 or CD68, using the Iba1 antibody as the initial marker in the staining sequence, followed by the 6E10 or CD68 antibody. Next, three sections for each Iba1/6E10- (−1.46 mm, −2.18 mm and −3.28 mm from the bregma) and Iba1/CD68-stained tissue (−1.46 mm, −2.06 mm and −2.46 mm from the bregma) were analysed using Stereo Investigator v. 9.10.6 (MBF Bioscience, MicroBrightField Inc., Williston, VT). For each section, amyloid load (number and area), microglia number and CD68 marking (number and area) were determined per hemisphere and expressed as ratios of the hippocampus area. Pictures were taken with a Nikon C80i microscope (Nikon Instruments, Williston, VT) equipped with a QImaging® color camera (MBF 2000 R, Quantitative Imaging, Surrey, BC) and QCapture Version 2.98.2 software (Quantitative Imaging).

### Western blot analysis

Whole brain homogenates were diluted 1:10 in NET lysis buffer (0.2 M NaCl, 0.1 M Tris, 5 mM EDTA, 11.6 mg/L Tergitol® solution (232mg/L) type NP-40, pH = 7.5) containing 1% (v/v) of phosphatase inhibitor mixture and 1% (v/v) of protease inhibitor cocktail (Sigma-Aldrich) and sonicated for 40 sec on ice (Sonic Dismembrator, model 100, Fisher Scientific Ca., Ottawa, ON). Total protein content for each sample was determined using the bicinchoninic acid method (QuantiPro assay kit, Sigma-Aldrich) [60]. Protein samples (15 μg) were mixed with 2X SDS loading buffer and heated for 5 min at 100°C. Samples were loaded on a precast 4-20% polyacrylamide gradient gel (Bio-Rad, Hercules, CA) and subjected to electrophoresis for 10 min at 110 V followed by 90 min at 90 V. After migration, resolved protein bands were transferred onto a 0.45-mm polyvinylidene fluoride (PVDF) membrane (EMD Millipore) for one hour on ice under a 80 V potential. The PVDF membrane was rinsed three times with a 0.1 M Tris-buffered saline containing 670 M Tween-20 (TBS-Tween; Sigma-Aldrich) and blocked in TBS-Tween with 5% (w/v) skim milk for 30 min at RT. The PVDF membrane was then incubated overnight at 4°C one of the following primary antibodies: anti-phosphoERK1/2 (T202/Y204 for ERK1, T185/Y187 for ERK2; Cell Signaling Technology, Danvers, MA), anti-total ERK1/2 (Cell Signaling Technology), anti-phospho-p38 (New England Biolabs, Whitby, ON), anti-total p38 (New England Biolabs), anti-phospho-stress-activated protein kinase/Jun amino-terminal kinase (SAPK/JNK; New England Biolabs), anti-total SAPK/JNK (New England Biolabs), anti-claudin-5 (Santa Cruz Biotechnology, Dallas, TX) and anti-β-actin (EMD Millipore), the latter being used as an internal standard for sample processing errors. The membrane was washed three times with TBS-tween, incubated for two hours at 4°C with the horseradish peroxidase-conjugated secondary antibody in blocking solution, and re-washed three times with TBS-tween. Proteins were detected by enhanced chemiluminescence (ECL; GE Healthcare Life Sciences, Mississauga, ON) [61] and exposed on BioMax® MR film (Carestream, Rochester, NY). Blots were digitized and analyzed by densitometry with ImageJ software (National Institutes of Health, Bethesda, MD). Specific protein levels were expressed relative to the loading control (β-actin internal standard).

### Flow cytometry

Blood collected in EDTA-coated vials was used to analyze monocytes in peripheral blood using flow cytometry. For this purpose, 50 μL of total blood was incubated on ice for 15 minutes with 4 μL purified rat anti-mouse CD16/CD32 antibody (Mouse BD Fc Block; BD BioSciences) diluted in 35 μL Ca^2+^/Mg^2+^-free Dulbecco's PBS (DPBS; Sigma-Aldrich). Always on ice, the mixture of cells and anti-mouse CD16/CD32 was incubated with a cocktail of V500-conjugated anti-CD45 antibody (BD BioSciences), Alexa700-conjugated anti-CD11b antibody (eBioScience, San Diego, CA), allophycocyanin-conjugated anti-CD115 antibody (eBioScience), phycoerythrin-conjugated anti-Ly6-G antibody (BD Biosciences) and V450-conjugated anti-Ly6-C antibody (BD BioSciences). After a 40-min incubation, cells were rinsed with DPBS. Red blood cells were lysed with BD Pharm Lyse^TM^ (BD Biosciences) for 30 min at RT. Labeled cells were washed, resuspended in DPBS, and then injected in a flow cytometer (BD^TM^ LSR II, BD Biosciences). A minimum of 67,000 singlet events were acquired and analyzed with BD FACSDiva^TM^ software v.6.1.2 (BD Biosciences)

### Immunohistochemistry

Free floating brain sections were washed with KPBS three 10-min cycle, thus permeabilized and blocked for 20 min in KPBS containing 0.4% triton X-100, 1% bovine serum albumin and 4% serum of the secondary antibody's species (horse or goat). Goat anti-albumin antibody (Abcam, Cambridge, UK) and rat anti-CD45 antibody (BD Biosciences, San Jose, CA, USA) were incubated overnight in 0.5 X of the blocking solution. Free-floating brain sections were washed with KPBS three 10-min cycles and incubated for 2 h at RT with the appropriate secondary antibody, i.e. either biotinylated anti-goat antibody (Vector Laboratories, Burlingame, CA) or biotinylated anti-rat antibody (Vector Laboratories). Brain slices were washed, treated with avidin/biotin complex reagent (Vector Laboratories) for one hour at RT and washed again. Albumin and CD45 were stained in 0.5 g/L diaminobenzidine (DAB; Sigma-Aldrich) solution supplemented with 1.11 mM H_2_O_2_ for 8 min and 15 min, respectively. Sections were immediately washed, mounted onto SuperFrost slides, and dried overnight. Slides were dehydrated using a sequential treatment with 10 dips in H_2_O, 50% (v/v) EtOH, 70% (v/v) EtOH, twice in 95% (v/v) EtOH, thrice in 100% (v/v) EtOH, and finally twice in xylene. The dehydrated sections on slides were overlaid with coverslips using distyrene plasticizer xylene (DPX) mounting medium

IgG extravasation staining was performed as described above when used as a secondary antibody. Free-floating brain slices were washed and treated with a blocking solution (6.78 mM Triton X-100, 1% (w/v) BSA and 4% (v/v) horse serum). Biotinylated anti-mouse IgG antibody was incubated overnight at 4°C. Slices were rinsed with KPBS and immersed for 1 h with ABC mixture. Brain sections were washed, stained in DAB solution for 10 min, and re-washed. Slices were mounted onto slides, dried, dehydrated and overlaid with coverslips in DPX. Pictures were taken using a Nikon C80i microscope equipped with a QImaging® color camera.

### Nissl body staining

Brain sections were mounted on slides and dried overnight under vacuum. Slides were washed (2×10 min) in KPBS, and then fixed for 20 min in 4% (w/v) PFA. They were then dehydrated according to the following sequence: H_2_O, 50% (v/v) EtOH, 70% (v/v) EtOH, twice in 95% (v/v) EtOH, and thrice in 100% (v/v) EtOH; each dip carried out for 3 min), transferred next into xylene for 5, 30 and 2 min, and finally rehydrated using the reverse-order sequence (thrice in 100% (v/v) EtOH, twice in 95% (v/v) EtOH, and then 70% (v/v) EtOH, 50% (v/v) EtOH and H_2_O; 2 min for each dip). Slides were next dipped 20 times in 0.25% thionin (Sigma-Aldrich). Mounted brain slides were dehydrated again (20 dips in each of H_2_O, 50% (v/v) EtOH, and 70% (v/v) EtOH, and then 2 and 3 cycles of 3-min dips in 95% (v/v) and 100% (v/v) EtOH, respectively, and finally, 2 cycles of 3-min dips in xylene) before overlaying with coverslips in DPX mounting medium (Electron Microscopy Sciences).

For stereological analysis, 3 sections (−1.46, −2.06, and −2.46 mm from the bregma) were analyzed using the Stereo Investigator software. For each section, the cornu ammonis 1 through 3 (CA1/CA2, CA3) areas and the dentate gyrus area were defined and expressed as ratio of total hippocampus area for both hemispheres. Photographs were obtained with a Nikon C80i microscope equipped with a QImaging® color camera.

### Fluoro-jade b staining

FJB staining was used as an indicator of neuronal death as described [62]. After mounting brain sections onto slides and thorough overnight drying under vacuum, slices were fixed with 4% PFA for 20 min. Fixed slides were then rinsed twice with KPBS for 5 min, before a cycle of dehydration/rehydration according to this sequence: 3 min in 50% (v/v) EtOH, 1 min in 70% (v/v) EtOH, 3 min in 100% (v/v) EtOH, 1 min in 70% (v/v) EtOH, 1 min in 50% (v/v) EtOH and 1 min in H_2_O). Mounted slides were next treated for 10 min with 3.8 mM potassium permanganate (MP Biomedicals, Santa Ana, CA), rinsed for 1 min with H_2_O and then incubated in 6.05 μM FJB solution (EMD Millipore) containing 17.5 mM acetic acid and 2 μg/mL DAPI. Then, slides were washed (3 × 1-min rinses in H_2_O) and dried overnight. Slides were immersed in xylene (3 × 2-min dips) and overlaid with coverslips in Fluoromount-G anti-fading mounting medium. Images were obtained with a Nikon C80i microscope equipped with a QImaging® color camera.

### *In vitro* experiments

#### Cell culture

Immortalized murine microglial cells (BV2) were cultured at 37°C in a 5% CO_2_/95% air atmosphere in DMEM containing 10% (v/v) FBS, 4.5 g/L glucose, 6 mM L-glutamine, 100 U/mL penicillin and 100 μg/mL streptomycin. Cells were grown to 70-90% confluence and subjected to a maximum of 20 passages. All cell culture reagents were from Sigma-Aldrich.

### XTT assay

Cell viability was measured with the 2,3-bis-(2-methoxy-4-nitro-5-sulfophenyl)-2H-tetrazolium-5-carboxanilide (XTT) assay according to the manufacturer's procedure (Cell Signaling Technology). BV2 cells were plated at 1×10^4^ cells/well in 96-well microplates in DMEM supplemented with 10% (v/v) FBS and 4.5 g/L D-glucose. Cells were incubated overnight at 37°C before replacing medium with DMEM containing 1% (v/v) FBS and either 4.5 g/L D-glucose (DMEM-High) or 1.0 g/L D-glucose (DMEM-Low; *n* = 5 experiments, 4 replicates/treatment). For cell death controls, cells were exposed for 24 h to DMEM-High containing 0.75 mM H_2_O_2_). Fifty μL of XTT detection solution were added to each well and incubated for 3 h at 37°C. Cell viability was determined from *A*_450_ using a microtiter plate reader and analyzed with the SOFTmax Pro3.1.1 software.

### Griess assay

Global cell activity was evaluated by determining nitrite release into the cell culture medium using the Griess assay according to the manufacturer's protocol (Life Technologies). Cells (1.8 × 10^4^/well) were plated in 24-well culture plates and incubated overnight at 37°C. BV2 cells were incubated for 24 h in DMEM-High or -Low medium plus or minus lipopolysaccharide (LPS) (Sigma-Aldrich) (at either 0.1, 0.5 or 1 μg/mL; *n* = 6 experiments, treatments in triplicate). Culture medium was then collected, and 150 μL were transferred to 96-well microplates. Nitrite-containing medium was mixed with 20 μL of Griess reagent and 130 μL of deionized water. Standards (1-100 μM) were added to the microplate, which was next incubated for 30 min at RT. Nitrite concentration was determined by measuring A_508_ with a microtiter plate reader and analyzed with the SOFTmax Pro3.1.1 software.

### CD68 expression analysis by flow cytometry

BV2 cells were plated (3×10^5^ cells/well) in 24-well culture plates in DMEM containing 10% (v/v) FBS and 4.5 g/L D-glucose. Cells were allowed to grow overnight at 37°C with 5% CO_2_ before replacing medium with DMEM-High or -Low (*n* = 5 for each group). After a 24-h incubation, cells were harvested upon treatment with cell dissociation buffer (Sigma-Aldrich) and transferred to 5-mL FACS tubes (BD Biosciences). Cells were spun down for 10 min at 300 x *g* and resuspended in DPBS containing 4% rat serum (Jackson ImmunoResearch). Cell metabolism was stopped by storage for 20 min on ice, and cells were then collected by centrifugation, washed and further incubated in 0.5 g/L Fc Block CD16/CD32 on ice for 20 min. Cells were next washed once with DPBS and suspended in 100 μL of DPBS containing 0.5 g/L Alexa 647-conjugated anti-CD68 antibody (AbD Serotec), and incubated on ice for 30 min. After one rinsing with DPBS, cells were resuspended in DPBS. Data were acquired using a flow cytometer (at least 10,000 singlet events) and analyzed with BD FACS Diva software.

### Phagocytic capacity by flow cytometry

BV2 cells were plated at 3×10^5^ cells per well in DMEM containing 4.5 g/L D-glucose and 10% (v/v) FBS, and incubated at 37°C overnight. DMEM was next replaced with DMEM-High or -Low (*n* = 6 for each group). Cells were acclimatized in the latter medium for 24 h before adding 5 mg/L of Vybrant *E. coli* beads (Life Technologies) in 1X Hank's balanced salts solution. Phagocytosis was allowed to proceed for 2 h at 37°C. As a negative control, phagocytosis was assessed on ice with viable cells. Cells were then washed twice with ice-cold DPBS and harvested with non-enzymatic cell dissociation buffer (Sigma-Aldrich). Non-internalized beads were quenched with 0.2% trypan blue solution (Sigma-Aldrich). BV2 cells were washed and resuspended in DPBS, and then injected into the flow cytometer. Data were acquired (at least 500 singlet events) and analyzed with the BD FACS Diva software.

### Statistical analysis

*In vivo* and *in vitro* data are expressed as the mean ± SEM and mean ± SD, respectively. For cellular and molecular analyses, comparisons between groups were analyzed with standard two-tailed unpaired *t*-tests. For weight variation through time, the intergroup differences were calculated by two-way ANOVA. Differences were considered as significant with a *P* value < 0.05. All statistical analyses were performed using GraphPad Prism v.6.0 for Windows (GraphPad Software Inc., La Jolla, CA).

## SUPPLEMENTARY MATERIAL FIGURES



## References

[1] Wimo A, Jonsson L, Winbald B (2006). An estimate of the worldwide prevalence and direct loss costs of dementia in 2003. Dement Geriatr Cogn Disord.

[2] Alzheimer A, Stelzmann RA, Schnitzlein HN, Murtagh FR (1995). An english translation of Alzheimer's 1907 paper, ‘Uber eine eigenartige erkankaung der hirnrinde’. Clin Anat.

[3] Iadecola C, Gorelick PB (2003). Converging pathogenic mechanisms in vascular and neurodegenerative dementia. Stroke.

[4] Knopman DS, Roberts R (2010). Vascular risk factors: Imaging and Neuropathologic correlates. J Alzheimers Dis.

[5] Marchesi VT (2011). Alzheimer's dementia begins as a disease of small blood vessels, damaged by oxidative-induced inflammation and dysregulated amyloid metabolism: Implication for early detection and therapy. FASEB J.

[6] Sagare AP, Bell RD, Zlokovic BV (2012). Neurovascular dysfunction and faulty amyloid β-peptide clearance in Alzheimer disease. Cold Spring Harb Perspect Med.

[7] de la Torre JC (2012). Cardiovascular risk factors promote brain hypoperfusion leading to cognitive decline and dementia. Cardiovasc Psychiatric Psychiatry Neurol.

[8] Kalaria RN, Akinyemi R, Ihara M (2012). Does vascular pathology contribute to Alzheimer changes?. J Neurol Sci.

[9] Cechetti F, Pagnussat AS, Worm PV, Elsner VR, Ben J, da Costa MS, Mestriner R, Weis SN, Netto CA (2012). Chronic brain hypoperfusion causes early glial activation and neuronal death, and subsequent long-term memory impairment. Brain Res Bull.

[10] Farkas E, Luiten PG (2001). Cerebral microvascular pathology in aging and Alzheimer's disease. Prog Neurobiol.

[11] Lee JS, Im DS, An YS, Hong JM, Gwag BJ, Joo IS (2011). Chronic cerebral hypoperfusion in a mouse model of Alzheimer's disease: An additional contribting factor of cognitive impairments. Neurosci Lett.

[12] Luckhaus C, Flüb MO, Wittsack HJ, Grass-Kapanke B, Jänner M, Khalili-Amiri R, Friedrich W, Supprian T, Gaebel W, Mödder U, Cohnen M (2008). Detection of changed regional cerebral blood flow in mild cognitive impairment and early Alzheimer's dementia by perfusion-weighted magnetic resonance imaging. NeuroiImage.

[13] Minoshima S, Giordani B, Berent S, Frey KA, Foster NL, Kuhl DE (1997). Metabolic reduction in the posterior cingulate cortex in very early Alzheimer's disease. Ann Neurol.

[14] Niwa K, Kazama K, Youkin SG, Carlson GA, Iadecola C (2002). Alterations in cerebral blood flow and glucose utilization in miceoverexpressing the amyloid precursor protein. Neurobiol Dis.

[15] Ruitenberg A, den Hejier T, Bakker SL, van Swieten JC, Koudstaal PJ, Hofman A, Breteler MM (2005). Cerebral hypoperfusion and clinical onset of dementia: the Rotterdam study. Ann Neurol.

[16] Zlokovic BV (2011). Neurovascular pathways to neurodegeneration in Alzheimer's disease and other disorders. Nat Rev Neurosci.

[17] Koike MA, Green KN, Blurton-Jones M, LaFerla FM (2010). Oligemic hypoperfusion differentially affects tau and amyloid-β. Am J Pathol.

[18] Okamoto Y, Yamamoto T, Kalaria RN, Senzaki H, Maki T, Hase Y, Kitamura A, Washida K, Yamada M, Ito H, Tomimoto H, Takahashi R, Ihara M (2012). Cerebral hypoperfusion accelerates cerebral amyloid angiopathy and promoter cortical microinfarcts. Acta Neuropathol.

[19] Bernstein SL, Dupuis NF, Lazo ND, Wyttenbach T, Condron MM, Bitan G, Teplow DB, Shea JE, Ruotolo BT, Ronbinson CV, Bowers MT (2009). Amyloid-β protein oligomerization and the importance of tetramers and dodecamers in the aetiology of Alzheimer's disease. Nat Chem.

[20] Iadecola C (2004). Neurovascular regulation in the normal brain and in Alzheimer's disease. Nat Rev Neurosci.

[21] Zlokovic BV (2008). The blood-brain barrier in health and chronic neurodegenerative disorders. Neuron.

[22] Kuchibhotla KV, Lattarulo CR, Hyman BT, Bacskai BJ (2009). Synchronous hyperactivity and intercellular calsium waves in astrocytes in Alzheimer mice. Science.

[23] Peppiatt CM, Howarth C, Mobbs P, Attwell D (2006). Bidirectional control of CNS capillary diameter by pericytes. Nature.

[24] Takano T, Han X, Deane R, Zlokovic BV, Nedergaard M (2007). Two-Photon Imaging of Astrocytic Ca2+ Signaling and the Microvasculature in Experimental Mice Models of Alzheimer's Disease. Ann N Y Acad Sci.

[25] ElAli A, Thériault P, Préfontaine P, Rivest S (2013). Mild chronic cerebral hypoperfusion induces neurovascular dysfunction, triggering peripheral beta-amyloid brain entry and aggregation. Acta Neuropathol Commun.

[26] Toyama K, Koibuchi N, Uekawa K, Hasegawa Y, Kataoka K, Katayama T, Sueta D, Ma MJ, Nakagawa T, Yasuda O, Tomimoto H, Ichijo H, Ogawa H (2014). Apoptosis signal-regulating kinase 1 is a novel target molecule for cognitive impairment induced by chronic cerebral hypoperfusion. Arterioscler Thromb Vasc Biol.

[27] Reimer MM, McQueen J, Searcy L, Scullion G, Zonta B, Desmazieres A, Holland PR, Smith J, Gliddon C, Wood ER, Herzyk P, Brophy P, McCulloch J (2011). Rapid disruption of axon-glial integrity in response to mild cerebral hypoperfusion. J Neurosci.

[28] Lana D, Melani A, Pugliese AM, Cipriani S, Nosi D, Pedata F, Giovannini MG (2014). The neuron-astrocyte-microglia triad in a rat model of chronic cerebral hypoperfusion: Protective effect of dipyridamole. Front Aging Neurosci.

[29] Jing Z, Shi C, Zhu L, Xiang Y, Chen P, Xiong Z, Li W, Ruan Y, Huang L (2015). Chronic cerebral hypoperfusion induces vascular plasticity and hemodynamics but also neuronal degeneration and cognitive impairment. J Cereb Blood Flow Metab.

[30] Liu J, Jin DZ, Xiao L, Zhu XZ (2006). Paeoniflorin attenuates chronic cerebral hypoperfusion-induced learning dysfunction and brain damage in rats. Brain Res.

[31] Ni J, Matsumoto K, Li HB, Murakami Y, Watanabe H (1995). Neuronal damage and decrease of central acetylcholine level following permanent occlusion of bilateral common carotid arteries in rat. Brain Res.

[32] Farkas E, Institóris Á, Domoki F, Mihály A, Luiten PG, Bari F (2004). Diazoxide and dimethyl sulphoxide prevent cerebral hypoperfusion-related learning dysfunction and brain damage after carotid artery occlusion. Brain Res.

[33] Pimentel-Coelho PM, Michaud JP, Rivest S (2013). Effects of mild chronic cerebral hypoperfusion and early amyloid pathology on spatial learning and the cellular innate immune response in mice. Neurobiol Aging.

[34] Rivest S (2009). Regulation of innate immune response in the brain. Nat Rev Immunol.

[35] ElAli A, Bordeleau M, Thériault P, Filali M, Lampron A, Rivest S (2015). Tissue-plasminogen activator attenuates Alzheimer's disease-related pathology development in APPswe/PS1 mice. Neuropsychophamacology.

[36] Hickman SE, Allison EK, El Khoury J (2008). Microglial dysfunction and defective β-amyloid clearance pathways in aging Alzheimer's disease mice. J Neurosci.

[37] Cavanaugh JE, Ham J, Hetman M, Poser S, Yan C, Xia Z (2011). Differential regulation of mitogen-activated protein kinases ERK1/2 and ERK5 by neurotrophins, neuronal activity, and cAMP in neurons. J Neurosci.

[38] Filali M, Lalonde R, Rivest S (2009). Cognitive and non-cognitive behaviors in an APPswe/PS1 bigenic model of Alzheimer's disease. Genes Brain Behav.

[39] Ennaceur A, Delacour J (1988). A new one-trial test for neurobiological studies of memory in rats. 1: Behavioral data. Behav Brain Res.

[40] Lue LF, Kuo YM, Roher AE, Brachova L, Shen Y, Sue L, Beach T, Kurth JH, Rydel RE, Rogers J (1999). Soluble amyloid beta peptide concentration as a predictor of synaptic change in Alzheimer's disease. Am J Pathol.

[41] Lehmann J, Härtig W, Seidel A, Füldner C, Hobohm C, Grosche J, Krueger M, Michalski D (2014). Inflammatory cell recruitment after experimental thromboembolic stroke in rats. Neuroscience.

[42] Urra X, Villamor N, Amaro S, Gómez-Choco M, Obach V, Oleaga L, Planas AM, Chamorro A (2009). Monocyte subtypes predict clinical course and prognosis in human stroke. J Cereb Blood Flow Metab.

[43] Michaud JP, Bellavance MA, Préfontaine P, Rivest S (2013). Real-time *in vivo* imaging reveals the ability of monocytes to clear vascular amyloid beta. Cell Reports.

[44] Naert G, Rivest S (2012). Hematopoietic CC-chemokine receptor 2 (CC2) competent cells are protective for the cognitive impairments and amyloid pathology in a transgenic mouse model of Alzheimer's disease. Mol Med.

[45] Naert G, Rivest S (2011). CC chemokine receptor 2 deficiency aggravates cognitive impairments and amyloid pathology in a transgenic mouse model of Alzheimer's disease. J Neurosci.

[46] Linch SN, Danielson ET, Kelly AM, Tamakawa RA, Lee JJ, Gold JA (2012). Interleukin 5 is protective during sepsis in an eosinophil-independent manner. Am J Respir Crit Care Med.

[47] Takeda Y, Costa S, Delamarre E, Roncal C, Leite de Oliveira R, Squadrito ML, Finisguerra V, Deschoemaeker S, Bruyere F, Wenes M, Hamm A, Serneels J, Magat J (2011). Macrophage skewing by Phd2 haplodeficiency prevents ischaemia by inducing arteriogenesis. Nature.

[48] Sugiyama Y, Yagita Y, Oyama N, Terasaki Y, Omura-Matsuoka E, Sasaki T, Kitagawa K (2011). Granulocyte colony-stimulating factor enhances arteriogenesis and ameliorates cerebral damage in a mouse model of ischemic stroke. Stroke.

[49] Michael OO, Gbolahan BW, Ansa CE, Abdulbasitand A, Azeez IO (2015). Endothelial proliferation modulates neuron-glia survival and differentiation in ischemic stress. Ann Neurosci.

[50] Nahrendorf M, Swirski FK, Aikawa E, Stangenberg L, Wurdinger T, Figueiredo JL, Libby P, Weissleder R, Pittet MJ (2007). The healing myocardium sequentially mobilizes two monocytes subsets with divergent and complementary functions. J Exp Med.

[51] Zhang RL, Zhang ZG, Chopp M (2007). Gene profile within the adult subventricular zone niche: proliferation, differenciation and migration of neural progenitor cells in the ischemic brain. Curr Mol Med.

[52] Stampfer MJ (2006). Cardiovascular disease and Alzheimer's disease: common links. J Intern Med.

[53] Yang G, Kitagawa K, Matsushita T, Yagita Y, Yanagihara T, Matsumoto M (1997). C57BL/6 strain is most susceptible to cerebral ischemia following bilateral common carotid occlusion among seven mouse strains: selective neuronal death in the murine transient forebrain ischemia. Brain Res.

[54] Yamada M, Ihara M, Okamoto Y, Maki T, Washida K, Kitamura A, Hase Y, Ito H, Takao K, Miyakawa T, Kalaria RN, Tomimoto H, Takahashi R (2011). The influence of chronic cerebral hypoperfusion on cognitive function and amyloid β metabolism in APP overexpressing mice. PLoS One.

[55] Gliem M, Mausberg AK, Lee JI, Simiantonakis I, van Rooijen N, Hartung HP, Jander S (2012). Macrophages prevent hemorrhagic infarct transformation in murine stroke models. Ann Neurol.

[56] Michaud JP, Pimentel-Coelho PM, Tremblay Y, Rivest S (2014). The impact of Ly6Clow monocytes after cerebral hypoxia-ischemia in adult mice. J Cereb Blood Flow Metab.

[57] Erecińska M, Silver IA (1989). ATP and brain function. J Cereb Blood Flow Metab.

[58] Gimeno-Bayón J, López-López A, Rodríguez MJ, Mahy N (2014). Glucose pathways adaptation supports acquisition of activated microglia phenotype. J Neurosci Res.

[59] Filali M, Lalonde R, Rivest S (2011). Anomalies in social behaviors and exploratory activities in an APPswe/PS1 mouse model of Alzheimer's disease. Physiol Behav.

[60] Smith PK, Krohn RI, Hermanson GT, Mallia AK, Gartner FH, Provenzano MD, Fujimoto EK, Goeke NM, Olson BJ, Klenk DC (1985). Measurement of protein using: bicinchoninic acid. Anal Biochem.

[61] Thorpe GH, Kricka LJ (1986). Enhanced chemiluminescent reactions catalyzed by horseradish perodish peroxidase. Methods Enzymol.

[62] Turrin NP, Rivest S (2006). Tumor necrosis factor alpha but not interleukin 1 beta mediates neuroprotection in response to acute nitric oxide excitotoxicity. J Neurosci.

