# Supplementary material for: Severe chronic cerebral hypoperfusion induces microglial dysfunction leading to memory loss in APPswe/PS1 mice
Source: Oncotarget. 2016 Feb 24;7(11):11864–80. doi: 10.18632/oncotarget.7689 (PMC4914254; doi:10.18632/oncotarget.7689)
Supplement: Supplementary file 1 [file oncotarget-07-11864-s001.pdf]

Severe chronic cerebral hypoperfusion induces microglial dysfunction leading to memory loss in APPswe/PS1 mice

Supplementary Material

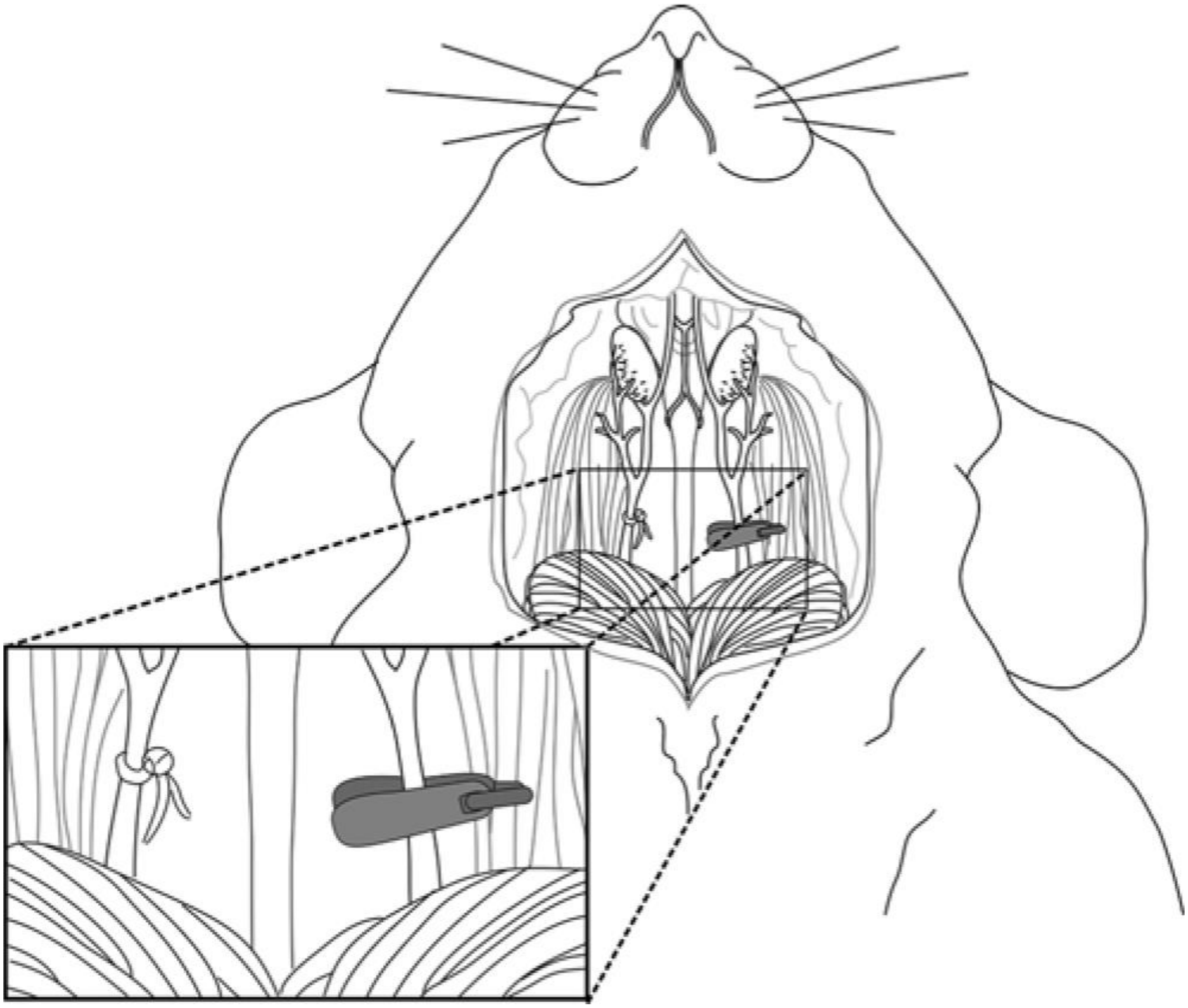

Supplementary Fig. 1: Scheme illustrating the SCCH surgery. Permanent occlusion of the right common carotid artery is performed in anesthetized mice. After 2 minutes, a 15G pressure is exerted with a vessel clip on the left common carotid artery during 15 seconds.

A)

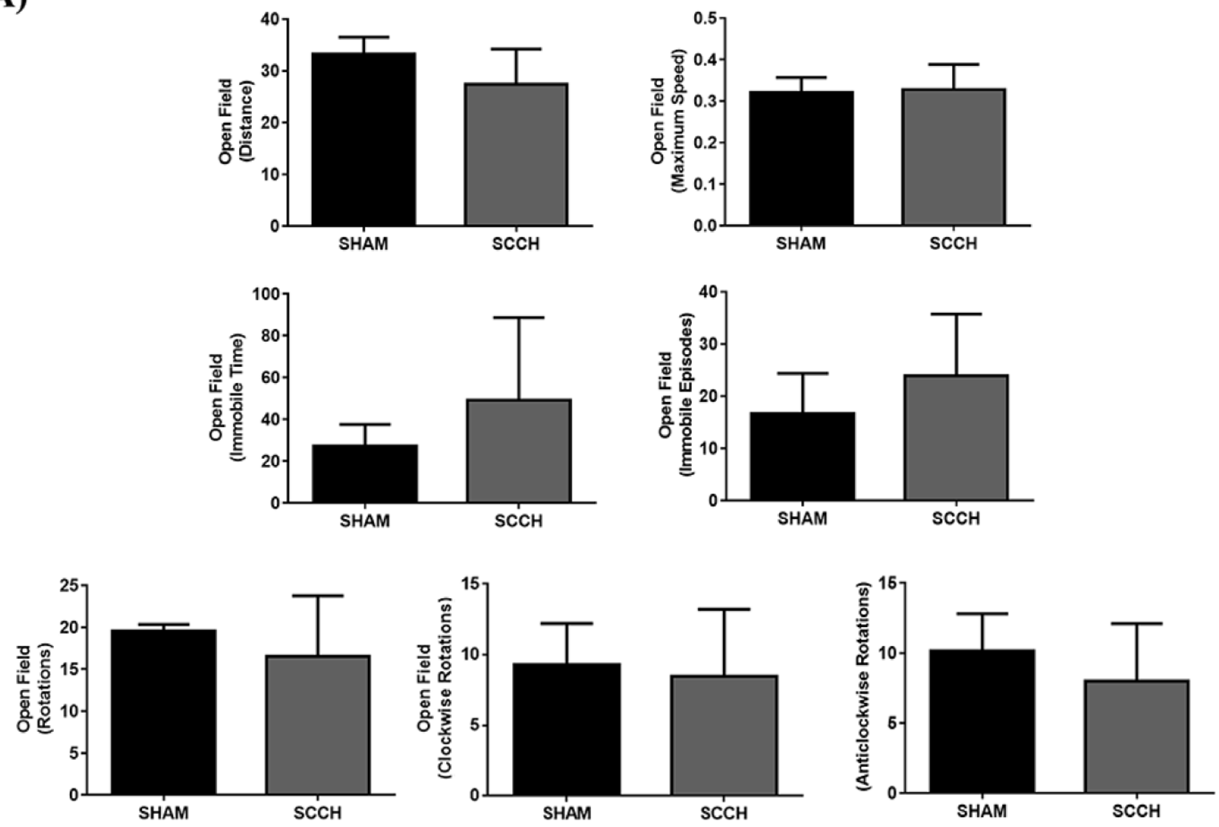

B)

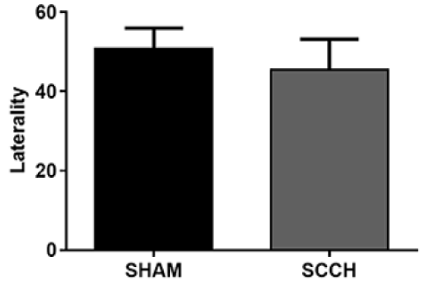

Supplementary Fig. 2: Motor performences in APPswe/PS1 after SCCH. Motor performances were assessed by using open field test **A.** and asymmetry cylinder test **B.** which define motor capacity and lateral asymmetry, respectively. SCCH does not induce motor deficits in mice **A., B**. Data are means ± SEM (n=6-8 animals per group). Data were analyzed with standard two-tailed unpaired *t*-test.

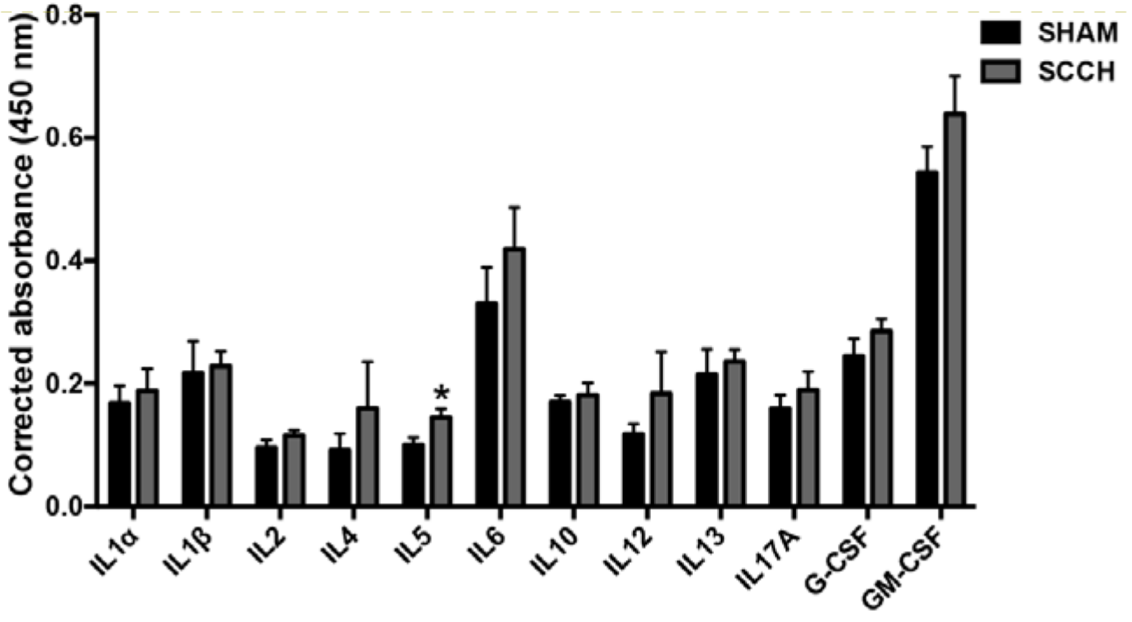

Supplementary Fig. 3: SCCH increases the protein expression level of IL5 in serum. The expression level of key cytokines in the serum of mice was assessed by ELISA assay. IL5 protein expression level significantly increases in the serum of SCCH mice. Data are means ± SEM (n=3 pooled serum samples from 6 animals). * $P < 0.05$ compared to sham Data were evaluated with standard two-tailed unpaired *t*-test.

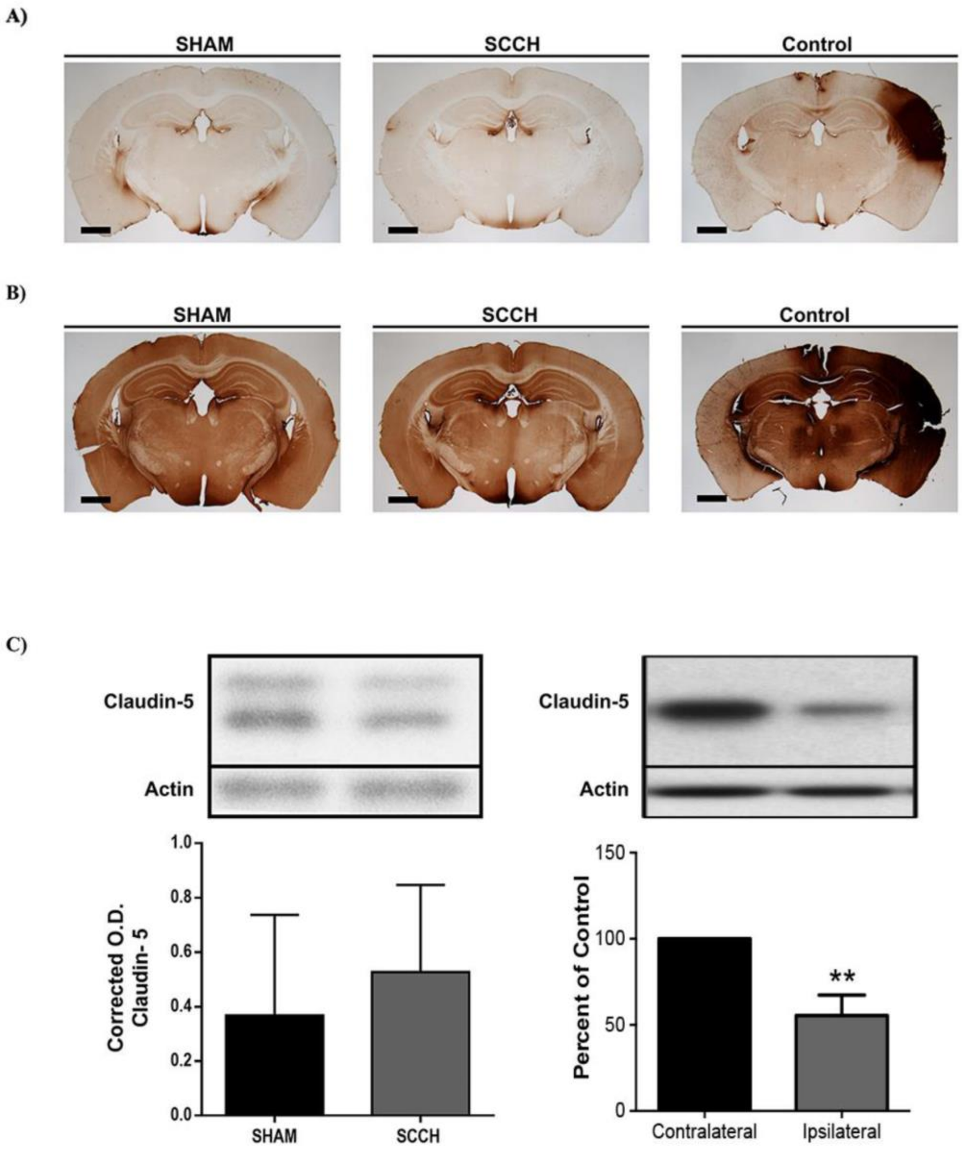

Supplementary Fig. 4: SCCH does not alter BBB tightness. BBB leakage is shown by immunocytochemistry **A, B**. Claudin V level is quantified by Western blot **C.** and corrected with β-actin expression level. SCCH does not induce IgG **A.** and albumin **B.** extravasation, and does not alter claudin-5 expression **C.**. Positive control demonstrates IgG **A.** and albumin **B.** extravasation 48 hours after stroke. Positive control demonstrates claudin-5 decreased expression. Data are means ± SEM (n=5-7 animals per group). ** $P < 0.01$ compared to sham. Data were evaluated by standard two-tailed unpaired *t*-test. Images were acquired with 1X objective. Scale bar = 1mm

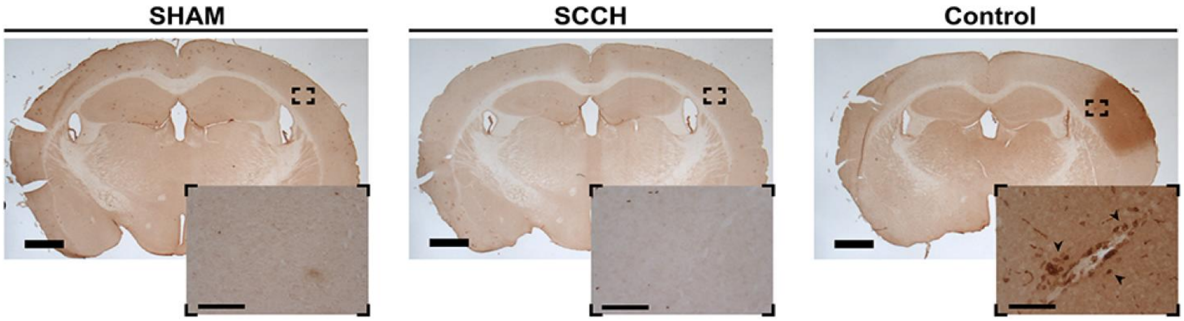

Supplementary Fig. 5: Absence of infiltrated leukocytes after SCCH. Leukocyte ($CD45^+$) infiltration is verified by immunocytochemistry. Both, sham and SCCH mice do not show leukocyte infiltration. Positive control shows leukocyte infiltration in the lesion site 48 hours after stroke. Images were acquired with 1X and 40X objectives. Scale bar = 1mm (1X objective); Scale bar = 75µm (40X objective).

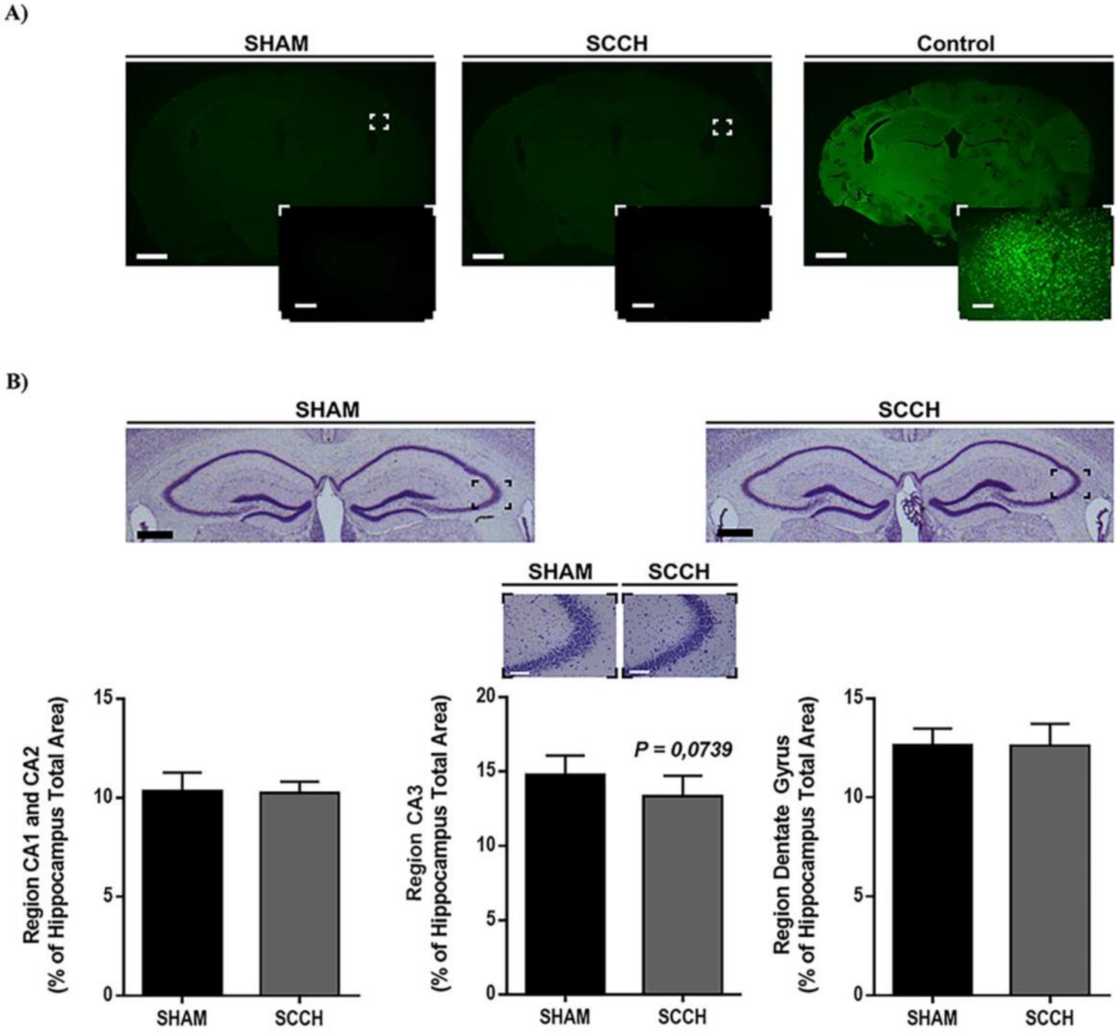

Supplementary Fig. 6: SCCH triggers a slight CA3 atrophy without neuronal death. Neuronal death and structural changes in hippocampus are analyzed by FJB **A.** and thionin staining **B.,** respectively. Positive controls present neuronal death at the lesion site 48 hours after stroke, whereas sham and SCCH mice do not **A.**. SCCH mice develop a slight (P = 0.0739) atrophy of CA3 region, without affecting CA1, CA2 and, dentate gyrus regions **B.**. Data are means ± SEM. Data are analyzed by standard two-tailed unpaired *t*-test. FJB staining images were acquired with 1.25X and 20X objectives. Thionin staining images were acquired with 1X and 20X objectives. Scale bar = 1mm (1.25X objective); Scale bar = 500µm (1X objective) ; Scale bar = 75µm (20X objective).
